# SARS‐CoV‐2 spike glycoprotein‐reactive T cells can be readily expanded from COVID‐19 vaccinated donors

**DOI:** 10.1002/iid3.496

**Published:** 2021-07-27

**Authors:** Pavla Taborska, Jan Lastovicka, Dmitry Stakheev, Zuzana Strizova, Jirina Bartunkova, Daniel Smrz

**Affiliations:** ^1^ Department of Immunology, Second Faculty of Medicine Charles University and University Hospital Motol Prague Czech Republic

**Keywords:** cellular immunity, COVID‐19 vaccination, ex vivo expansion, humoral immunity, SARS‐CoV‐2, spike glycoprotein‐reactive

## Abstract

**Introduction:**

The COVID‐19 vaccine was designed to provide protection against infection by the severe respiratory coronavirus 2 (SARS‐CoV‐2) and coronavirus disease 2019 (COVID‐19). However, the vaccine's efficacy can be compromised in patients with immunodeficiencies or the vaccine‐induced immunoprotection suppressed by other comorbidity treatments, such as chemotherapy or immunotherapy. To enhance the protective role of the COVID‐19 vaccine, we have investigated a combination of the COVID‐19 vaccination with ex vivo enrichment and large‐scale expansion of SARS‐CoV‐2 spike glycoprotein‐reactive CD4^+^ and CD8^+^ T cells.

**Methods:**

SARS‐CoV‐2‐unexposed donors were vaccinated with two doses of the BNT162b2 SARS‐CoV‐2 vaccine. The peripheral blood mononuclear cells of the vaccinated donors were cell culture‐enriched with T cells reactive to peptides derived from SARS‐CoV‐2 spike glycoprotein. The enriched cell cultures were large‐scale expanded using the rapid expansion protocol (REP) and the peptide‐reactive T cells were evaluated.

**Results:**

We show that vaccination with the SARS‐CoV‐2 spike glycoprotein‐based mRNA COVID‐19 vaccine‐induced humoral response against SARS‐CoV‐2 spike glycoprotein in all tested healthy SARS‐CoV‐2‐unexposed donors. This humoral response was found to correlate with the ability of the donors' PBMCs to become enriched with SARS‐CoV‐2 spike glycoprotein‐reactive CD4^+^ and CD8^+^ T cells. Using an 11‐day REP, the enriched cell cultures were expanded nearly 1000‐fold, and the proportions of the SARS‐CoV‐2 spike glycoprotein‐reactive T cells increased.

**Conclusion:**

These findings show for the first time that the combination of the COVID‐19 vaccination and ex vivo T cell large‐scale expansion of SARS‐CoV‐2‐reactive T cells could be a powerful tool for developing T cell‐based adoptive cellular immunotherapy of COVID‐19.

## Graphical abstract

The COVID‐19 vaccine was designed to provide protection against infection by SARS‐CoV‐2 and COVID‐19. Using a peptide‐based enrichment and rapid expansion protocol, we could generate large numbers of the SARS‐CoV‐2 spike glycoprotein‐reactive T cells from COVID‐19 vaccinated donors.

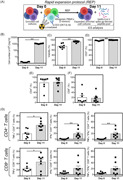

**Abbreviations**
COVID‐19: coronavirus disease 2019
PBMC: peripheral blood mononuclear cell
RBD: receptor‐binding domain
REP: rapid expansion protocol
SARS‐CoV‐2: severe respiratory coronavirus 2

## 1 INTRODUCTION

COVID‐19 is transforming into more severe and contagious forms as the severe respiratory coronavirus 2 (SARS‐CoV‐2) mutates during the pandemic.^1^ The recently appeared new mutations of the virus seem to evade the immune system more efficiently, including evasion of the coronavirus disease 2019 (COVID‐19) vaccine‐induced immunity.^2^ This evasion lies in the decreased capability of SARS‐CoV‐2‐specific antibodies to neutralize the virus efficiently.^3^ Since the antibody‐mediated protection depends on the structure of the target antigen, any mutation causing a productive conformational change of the target antigen can decrease the antibody binding and erode its protective role.^4^, ^5^ The current COVID‐19 vaccines are nearly exclusively targeting a single protein of the virus, the spike glycoprotein, so chances of evasion could be high.

The antiviral immune response also relies on adaptive cellular immunity where the antiviral effectors are, instead of antibodies, the cytotoxic CD8^+^ T cells which recognize infected cells expressing viral proteins.^6^ Unlike antibodies, the viral proteins are recognized in the form of protein fragments (peptides) presented in the context of the major‐histocompatibility complexes and T cell receptors (TCRs).^6^ Recent studies show that SARS‐CoV‐2 T cell‐based immunity is negligibly impacted by the current mutated variants of SARS‐CoV‐2^7^ and, therefore, could counteract the debilitating impact these mutations might have on the parallel humoral immunity.^8^ However, many immunocompromised patients, patients with immunodeficiencies, or patients with a comorbidity treatment‐suppressed immunity, such as patients undergoing chemotherapy or immunotherapy, may not sufficiently mobilize the cellular immunity against SARS‐CoV‐2 after vaccination. It is, therefore, necessary to find new ways to enhance their cellular immunity against the virus.

Previous studies have shown that SARS‐CoV‐2 T cell‐based immunity could be enhanced and used for T cell‐based therapy of COVID‐19 after ex vivo large scale expansion of SARS‐CoV‐2‐specific T cells from COVID‐19 convalescent donors.^9^, ^10^ Whether this enhancement could also be attained and therapeutically harvested after COVID‐19 vaccination of donors with no previous history of COVID‐19 and/or no detectable SARS‐CoV‐2‐specific immunity is unknown.

This study examined the impact of the COVID‐19 vaccine on the induction of the humoral and cellular responses in eight healthy donors who had no history of COVID‐19, were seronegative for SARS‐CoV‐2 antibodies, and showed no or minimal CD8^+^ T cell reactivity to SARS‐CoV‐2 spike glycoprotein‐derived peptides before the vaccination. We investigated whether their cellular response to the vaccine could be enhanced by the ex vivo enrichment and large‐scale expansion and hence represent an avenue for promoting the SARS‐CoV‐2‐specific cellular immunity in patients who could not fully benefit from the COVID‐19 vaccines.

## 2 MATERIALS AND METHODS

### 2.1 Donors and COVID‐19 vaccination

The study involved eight healthy donors who were negative for SARS‐CoV‐2 spike glycoprotein‐specific antibodies and who reported no previous history of COVID‐19 or positivity for SARS‐CoV‐2 infection. The median age of the donors was 46.0 years (range: 32–72 years) and the samples were obtained between October 2020 and February 2021. The donors were vaccinated with the BNT162b2 SARS‐CoV‐2 vaccine (Pfizer‐BioNTech) with two doses with a 3‐ to 4‐week interval between each dose. The donors' samples, the peripheral blood serum and unclotted peripheral blood, were collected up to 2 days before the first and second dose of the vaccine and 3–4 weeks after the second dose of the vaccine. The serum was separated by centrifugation at 3000 rpm for 5 min at room temperature and cryopreserved. PBMCs from the unclotted peripheral blood were isolated as previously described^11^ and cryopreserved (RPMI 1640 medium [Thermo Fisher Scientific], 10% human plasma serum [One Lambda], 10% DMSO [Sigma‐Aldrich], 100 U/ml penicillin‐streptomycin, and 2 mM GlutaMax [Thermo Fisher Scientific]). As the controls were used two donors (patients) whose samples were first collected before they contracted SARS‐CoV‐2 and became sick with COVID‐19, and then 2 weeks after their recovery from the disease. Each donor provided signed written informed consent for the use of their blood‐derived products for future research and all experimental protocols were approved by the ethical standards of the institutional research committee—the Ethics Committee of the University Hospital Motol in Prague, and performed in accordance with the 1964 Helsinki declaration and its later amendments or comparable ethical standards.

### 2.2 Microblot array

The collected donors' sera were analyzed for the presence of multiple antigen‐specific antibodies using Microblot‐Array COVID‐19 IgG, IgA, or IgM kits (TestLine Clinical Diagnostics). The analyses were performed according to the manufacturer's instructions. The levels of the specific antibodies were evaluated according to the manufacturer's instructions in (U/ml). The samples with (U/ml) values <185 were negative, between 185 and 210 borderlines, and >210 positive. The IgG, IgA, and IgM antibodies against the following antigens were determined: SARS‐CoV‐2 spike glycoprotein RBD and S2 domain (S2), SARS‐CoV‐2 NCP, EP, and PLP, Middle East respiratory syndrome‐related coronavirus (MERS‐CoV) spike glycoprotein S1 subunit (S1), SARS‐CoV NCP, human coronavirus 229E (HuCoV 229E) NCP, human angiotensin‐converting enzyme (ACE‐2).

### 2.3 Peptide‐mediated enrichment of the antigen‐reactive T cells

The cryopreserved PBMCs were reconstituted at the concentration 2 × 10^6^ cells/ml in a human plasma serum‐containing medium (RPMI 1640 medium, 5% human plasma serum [One Lambda], 100 U/ml penicillin‐streptomycin, 2 mM GlutaMax, 1 mM sodium pyruvate, and nonessential amino acid mix [Thermo Fisher Scientific]) supplemented with 10 IU/ml of IL‐2 (PeproTech). The reconstituted PBMCs were stimulated with 0.5 μg/ml concentration of pooled overlapping peptides spanning the whole molecule of SARS‐CoV‐2 spike glycoprotein (PepMix™ SARS‐CoV‐2 Spike Glycoprotein, cat.# PM‐WCPV‐S‐1, JPT Peptide Technologies), nucleocapsid protein (PepMix™ SARS‐CoV‐2 (NCAP), cat.# PM‐WCPV‐NCAP, JPT), or membrane protein (PepMix™ SARS‐CoV‐2 [VME1], cat.# PM‐WCPV‐VME, JPT).^12^, ^13^ The cells were then cultured for 12 days supplementing the cell cultures with fresh media and IL‐2 every 2–4 days. The 12‐day cell cultures with the peptide‐reactive cells were processed immediately or cryopreserved.

### 2.4 Cell stimulation, intracellular cytokine staining, and T cell phenotype

The cultured cells or isolated PBMCs were stimulated with 0.5 μg/ml concentration of the peptide pool. After 1 h of culture (37°C, 5% CO_2_), the cells were supplemented with brefeldin A solution (BioLegend) and cultured for 4 h. The samples stimulated with the peptide solvent alone (20% DMSO in PBS) were used as unstimulated controls. The cells were transferred to a V‐bottom 96‐well plate (Nalgene) and stained as described^14^ with live/dead fixable stain and the following antibodies: CD4‐PE‐Cy7 and CD8‐Alexa Fluor 700 (Exbio), CD3‐PerCP‐Cy5.5, TNF‐α‐APC, IFNγ‐PE (Becton Dickinson). For T cell phenotype analyses, the cells were stained with live/dead fixable stain and the following antibodies: CD4‐PE‐Cy7 and CD8‐Alexa Fluor 700 (Exbio), CD3‐PerCP‐Cy5.5 and TNF‐α‐APC (Becton Dickinson), and CD62L‐FITC and CD45RO‐PE (Exbio). The cells were analyzed by FACSAria II (Becton Dickinson) and the data were processed by FlowJo software (Tree Star). The frequency of reactive T cells was calculated as the difference between the frequency of the cytokine‐producing T cells of the vehicle‐stimulated sample and the peptide pool‐stimulated sample of the same donor.

### 2.5 Rapid expansion protocol

The REP was performed for the large‐scale expansion of the peptide‐enriched cell cultures.^15^ PBMCs isolated from buffy coats were used as feeder cells. The buffy coats were obtained from the Institute of Hematology and Blood Transfusion in Prague and each donor provided signed written informed consent to participate in the study. The isolated PBMCs from three donors were pooled and γ‐irradiated (64 Gy; Gammacell 3000 ELAN [Best Theratronics]). The irradiated PBMCs were combined with the peptide‐enriched cell cultures, 100 ng/ml of CD3‐specific antibody (OKT‐3; Miltenyi Biotec), and cultured in tissue culture flasks (TPP) for 11 days in the human plasma serum‐containing medium with 3000 IU/ml of IL‐2 (PeproTech). The cell cultures were supplemented with fresh media and IL‐2 every 2–4 days. The expanded cultures were processed immediately or cryopreserved.

### 2.6 Statistical analysis

The means of values ± *SEM* were calculated from the indicated sample size (*n*) using GraphPad Prism 6 (GraphPad Software) and the statistical significance (*p < .05, **p < .01, ***p < .001, ****p < .0001) between two groups of samples determined by Wilcoxon matched‐pair signed‐rank tests and between three or more groups the statistical significance was determined by matched‐pair one‐way ANOVA with Dunn's post test. The associations between two variables were assessed by Spearman's rank‐order correlation coefficient (*r*) and the statistical significance of the correlation (*p*) was determined. Graphical images were created with Biorender.com (accessed in March and April 2021).

## 3 RESULTS

### 3.1 COVID‐19 vaccination induces SARS‐CoV‐2 spike glycoprotein‐specific antibodies

**Figure 1:**
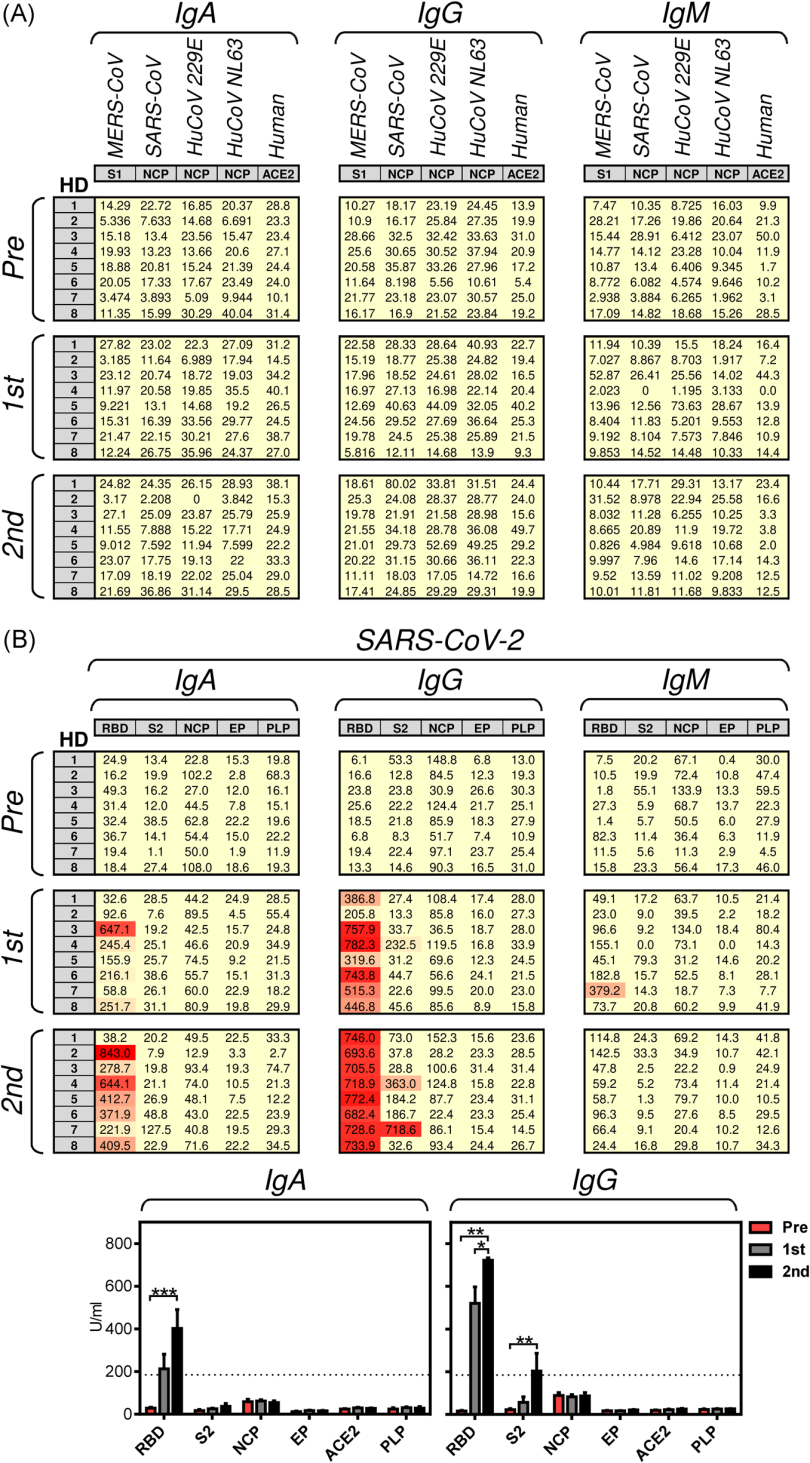
The serum levels of the antigen‐specific IgG, IgA, and IgM antibodies were determined by the Microblot‐Array COVID‐19. (A–B) The two vaccine doses were administered to eight healthy donors (HDs) with 3–4 weeks between the doses. The serum samples were collected 0–2 days before each vaccine dose (Pre, first), and 3–4 weeks after the second vaccine dose (second). In (A), the serum levels (U/ml) of IgA, IgG, and IgM antibodies specific to non‐SARS‐CoV‐2 proteins; Middle East respiratory syndrome‐related coronavirus spike glycoprotein S1 subunit (MERS‐CoV, S1), SARS‐CoV nucleocapsid protein (SARS‐CoV, NCP), human coronavirus 229E NCP (HuCoV 229E, NCP), human angiotensin‐converting enzyme (Human, ACE‐2). In (B), the serum levels (U/ml) of IgA, IgG, and IgM antibodies specific to SARS‐CoV‐2 proteins; spike glycoprotein receptor‐binding domain (RBD) and S2 domain (S2), nucleocapsid protein (NCP), E protein (EP), and papain‐like protease (PLP). The samples with (U/ml) values < 185 were negative, between 185 and 210 borderlines, and >210 positive. The data in (B) were evaluated (B, bottom panels). The bars represent the mean of values ± *SEM* and significances of differences among the groups (Pre, first, second) for individual proteins are indicated (*p < .05, **p < .01, ***p < .001, IgA: *n* = 7 [HD1 was excluded because diagnosed as IgA deficient] and IgG: *n* = 8 HDs, one‐way ANOVA with the Dunn's posttest)

We first investigated the humoral response of the mRNA SARS‐CoV‐2 spike glycoprotein‐based COVID‐19 vaccine, BNT162b2, in eight healthy donors who tested negative for the presence of antibodies specific to SARS‐CoV‐2 spike glycoprotein, who reported no previous history of COVID‐19. The donors were vaccinated with two doses of the vaccine within a 3‐ to 4‐week interval. Samples, the serum and unclotted blood, were collected during the 2 days before each vaccination and 3–4 weeks after the second dose of the vaccine. Using a microblot system, the sera were analyzed for the presence of SARS‐CoV‐2‐specific IgA, IgG, or IgM antibodies against the virus proteins: receptor‐binding domain (RBD) and S2 subunits of the spike glycoprotein (S2), nucleocapsid protein (NCP), envelope protein (EP), and papain‐like protease (PLP).^12^ As negative controls, the sera were analyzed for the presence of antibodies specific to human ACE‐2 protein^17^ or proteins from other coronaviruses: S1 subunit of the Middle East respiratory syndrome‐related coronavirus spike glycoprotein (MERS‐CoV),^18^ NCP of SARS‐CoV,^19^ human coronavirus 229E^20^ and NL63.^21^ As shown in Figure 1A, the COVID‐19 vaccination‐induced no production of SARS‐CoV‐2 unrelated antibodies. The COVID‐19 vaccination also did not induce the production of antibodies specific to the SARS‐CoV‐2 NCP, EP, or PLP (Figure 1B), which indicated no previous SARS‐CoV‐2 infection. On the other hand, the vaccine‐induced the production of antibodies specific to SARS‐CoV‐2 glycoprotein. As shown in Figure 1B (middle panel), the RBD‐specific IgG antibodies were already induced in all the tested donors after the first dose of the vaccine and their levels were further enhanced after the second dose of the vaccine. Only one donor after the first dose and two donors after the second dose of the vaccine produced IgG antibodies against the S2 subunit of the virus spike glycoprotein (Figure 1B, middle panels), indicating stronger immunogenicity of its RBD domain. This stronger immunogenicity was even more pronounced for the IgA‐specific antibodies where the vaccine‐induced the production of only the RBD‐specific IgA antibodies (Figure 1B, left panels). The production of these IgA antibodies was determined only in seven donors because one donor in the cohort was diagnosed IgA deficient. The production of IgM antibodies was detected only in one donor and only after the first dose of the vaccine (Figure 1B, right middle panel). The microblot results showed that the COVID‐19 vaccination predominantly induced a specific IgA or IgG antibody response against the RBD of the SARS‐CoV‐2 spike glycoprotein and less frequently a specific IgG antibody response against the S2 subunit of the SARS‐CoV‐2 spike glycoprotein.

### 3.2 COVID‐19 vaccination induces SARS‐CoV‐2 spike glycoprotein‐specific cellular immunity

**Figure 2:**
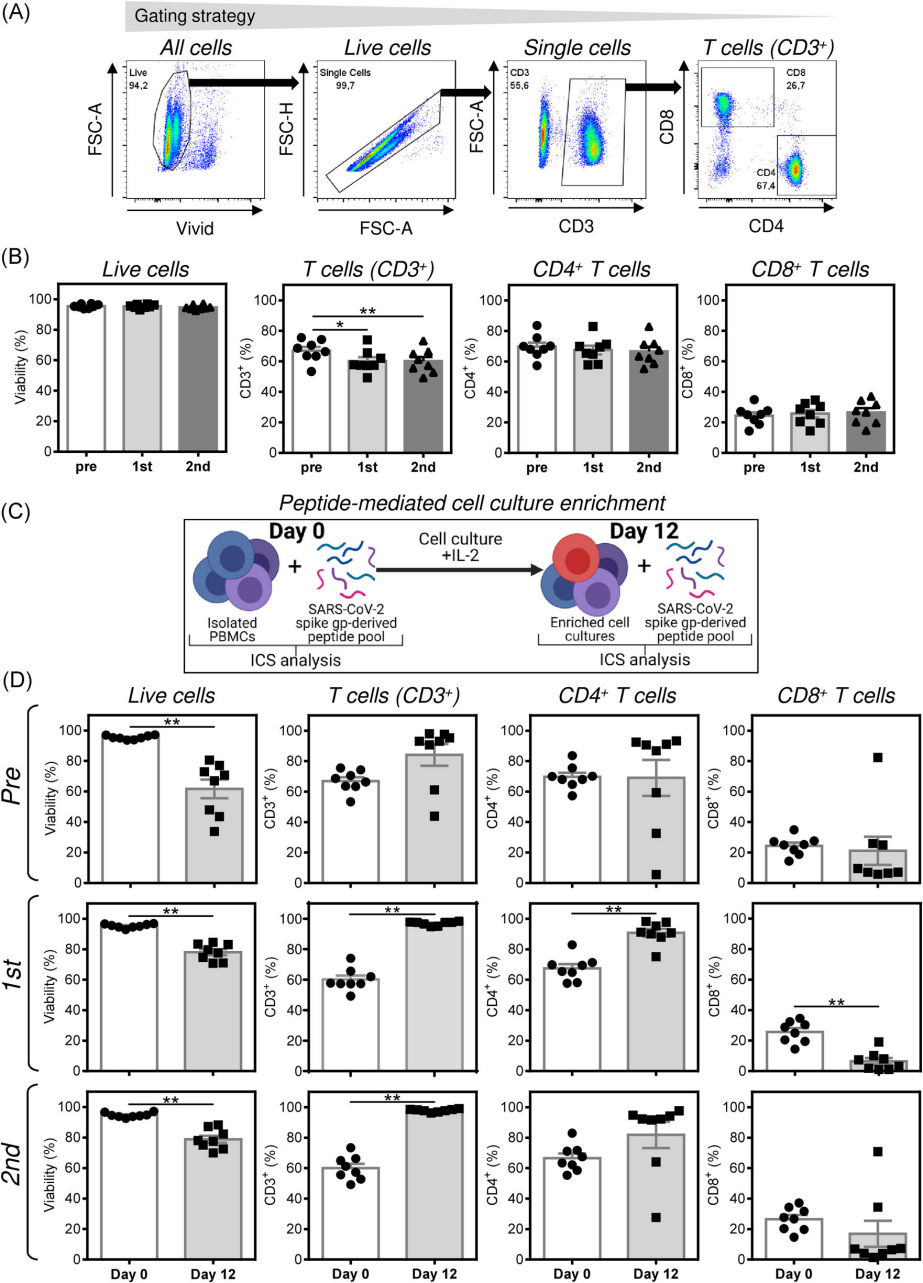
Characterization of the isolated PBMCs and peptide‐enriched cell cultures. (A, B) Isolated PBMCs from samples obtained as in Figure 1 before each vaccine dose (Pre, 1st), and 3–4 weeks after the second vaccine dose (2nd) were characterized by flow cytometry. In (A), the gating strategy of flow cytometry data. In (B), the cells were analyzed for their viability, the proportion of T cells (CD3^+^), and proportions of CD4^+^ and CD8^+^ populations of T cells. (C) Schematic presentation of the peptide‐mediated enrichment and intracellular cytokine staining (ICS) analyses. (D) PBMCs in (A) and (B) were peptide‐enriched for 12 days and analyzed as in (A) and (B). Their viability (Vivid^‐^), the proportion of T cells (CD3^+^), and proportions of CD4^+^ and CD8^+^ populations of T cells of the 12‐day peptide‐enriched cultures (Day 12) were determined, and the data compared with PBMCs before the enrichment (Day 0). In (B) and (D), the bars represent mean of values ± *SEM* and significances of differences between PBMCs (Day 0) and the peptide‐enriched cell cultures (Day 12) were determined for individual groups of the collected samples (Pre, 1st, 2nd; **p < .01, *n* = 8 healthy donors, Wilcoxon matched‐pairs signed‐ranks test). PBMC, peripheral blood mononuclear cell

The cellular immunity against SARS‐CoV‐2 is increasingly considered to be as important for the effective protection against the virus as the humoral immunity.^22^ Since our data showed that the COVID‐19 vaccine specifically induced humoral response against SARS‐CoV‐2 spike glycoprotein, we next investigated whether the COVID‐19 vaccination impacted the reactivity of the donors' CD4^+^ and CD8^+^ T cells to peptides derived from SARS‐CoV‐2 spike glycoprotein. We first found that the COVID‐19 vaccination did not affect the viability of the isolated donors' peripheral blood mononuclear cells (PBMCs; Figures 2A and 2B). The vaccination also had a minimal effect on the proportions of T cells and their CD4^+^ and CD8^+^ subpopulations (Figures 2A and 2B). To determine the reactivity of the donors' CD4^+^ and CD8^+^ T cells to peptides derived from the SARS‐CoV‐2 spike glycoprotein, the donors' PBMCs were stimulated with a pool of peptides derived from the glycoprotein (Figure 2C). The peptide pool‐stimulated cells were either analyzed by intracellular cytokine staining (ICS) after a 5 h stimulation or cultured for 12 days in the presence of IL‐2 to enrich the cell cultures for the peptide‐specific T cells.^23^ Following the stimulation of the 12‐day‐enriched cell cultures with the peptide pool for 5 h, the presence of the peptide‐specific T cells was determined by ICS (Figure 2C). As shown, the 12‐day cell culture enrichment decreased the viability of the cultured cells (Figure 2D, left panels) but increased the content of T (CD3^+^) cells in the samples obtained after the COVID‐19 vaccinations (Figure 2D, middle panels). The cell culture enrichment also altered the proportions of CD4^+^ and CD8^+^ populations of T cells in samples obtained after the first dose of the COVID‐19 vaccine (Figure 2D, two right‐hand panels in the second row).

**Figure 3:**
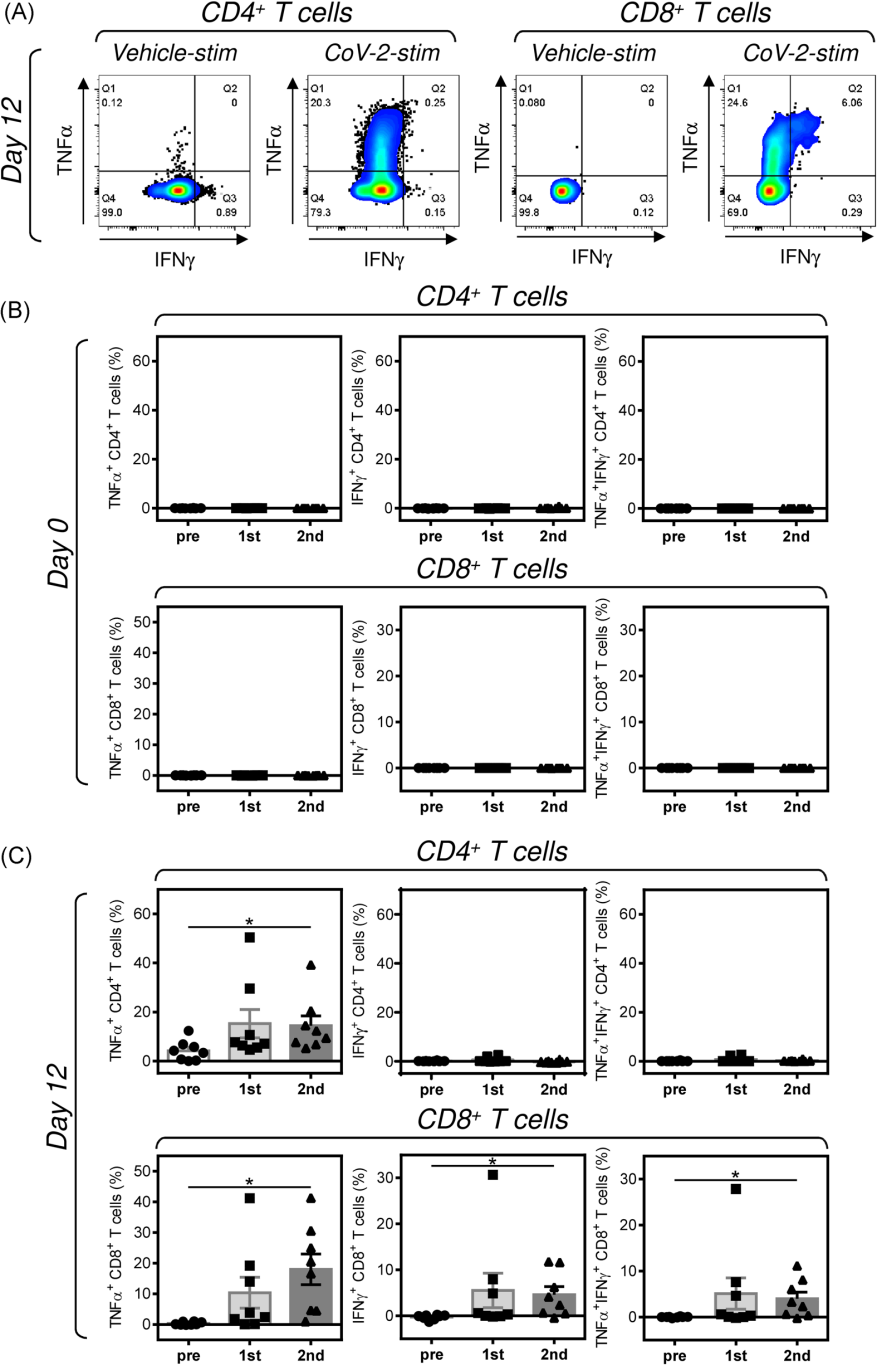
Reactivity of the isolated PBMCs and peptide‐enriched cell cultures to SARS‐CoV‐2 spike glycoprotein‐derived peptides. (A) The isolated PBMCs (Day 0) and peptide‐enriched cell cultures (Day 12) were (CoV‐2‐stim) or were not (Vehicle‐stim) stimulated with SARS‐CoV‐2 spike glycoprotein‐derived peptides and the proportions of TNF‐α‐, IFNγ‐, or TNF‐α/IFNγ‐producing CD4^+^ and CD8^+^ T cells determined by intracellular cytokine staining (ICS). The gating strategy of the flow cytometry data. (B, C) The proportions of reactive T cells in PBMCs (Day 0) (B) and the 12‐day peptide‐enriched cell cultures (Day 12) (C) were calculated as the difference between the proportions of the cytokine‐producing T cells of the vehicle‐stimulated sample and the peptide‐stimulated sample of the same donor. In (B) and (C), the bars represent the mean of values ± *SEM* and significances of differences among the groups (Pre, 1st, 2nd) for TNF‐α‐, IFNγ‐, or TNF‐α/IFNγ‐producing CD4^+^ and CD8^+^ T cells are indicated (*p < .05, *n* = 8 healthy donors, one‐way ANOVA with the Dunn's posttest). PBMC, peripheral blood mononuclear cell

The presence of peptide‐specific T cell populations was determined by ICS of TNF‐α‐ and IFNγ‐producing T cells (Figure 3A). As shown in Figure 3B, the isolated PBMCs from all donors and regardless of the COVID‐19 vaccination contained no detectable TNF‐α‐, IFNγ‐, or TNF‐α/IFNγ‐producing CD4^+^ or CD8^+^ T cell populations reactive to the SARS‐CoV‐2 spike glycoprotein‐derived peptides. However, the 12‐day peptide‐mediated enrichment significantly enriched cell cultures with the peptide‐reactive T cell populations (Figure 3C). As shown, the peptide‐enriched cell cultures already contained TNF‐α‐producing CD4^+^ T cell population reactive to the peptides, and this population was significantly higher in cell samples enriched after the second dose of the vaccine than in cell samples enriched before the vaccination (Figure 3C, top left panel). No effect of the vaccination on the enrichment with the peptide‐reactive IFNγ‐ or TNF‐α/IFNγ‐producing CD4^+^ T cells was observed because no such populations were detected in the 12‐day‐enriched cell cultures (Figure 3C, middle and right top panels). However, the vaccination had a robust impact on the enrichment of cell cultures with the peptide‐reactive CD8^+^ T cells. As shown in Figure 3C (bottom panels), the pre‐vaccination samples were not enriched with the peptide‐reactive CD8^+^ T cells, showing that the donors failed to attain a peptide‐mediated ex vivo enrichment with the SARS‐CoV‐2 spike glycoprotein‐reactive CD8^+^ T cells. This failure was overcome by the COVID‐19 vaccination because after the second dose of the vaccine, the cell cultures became significantly enriched with the peptide‐reactive CD8^+^ T cells (Figure 3C, bottom panels). Moreover, this reactivity was shown not only for the TNF‐α‐producing CD8^+^ T cells (Figure 3C, bottom left panel) but also for the IFNγ‐ or TNF‐α/IFNγ‐producing CD8^+^ T cells (Figure 3C, middle and right bottom panels). These data showed that the COVID‐19 vaccination significantly promoted the ability of the donors' PBMCs to become ex vivo enriched with the SARS‐CoV‐2 spike glycoprotein‐reactive CD4^+^ and CD8^+^ T cells.

### 3.3 COVID‐19 vaccination‐induced humoral response correlates with the SARS‐CoV‐2 spike glycoprotein peptide reactivity of the peptide‐enriched PBMCs

**Figure 4:**
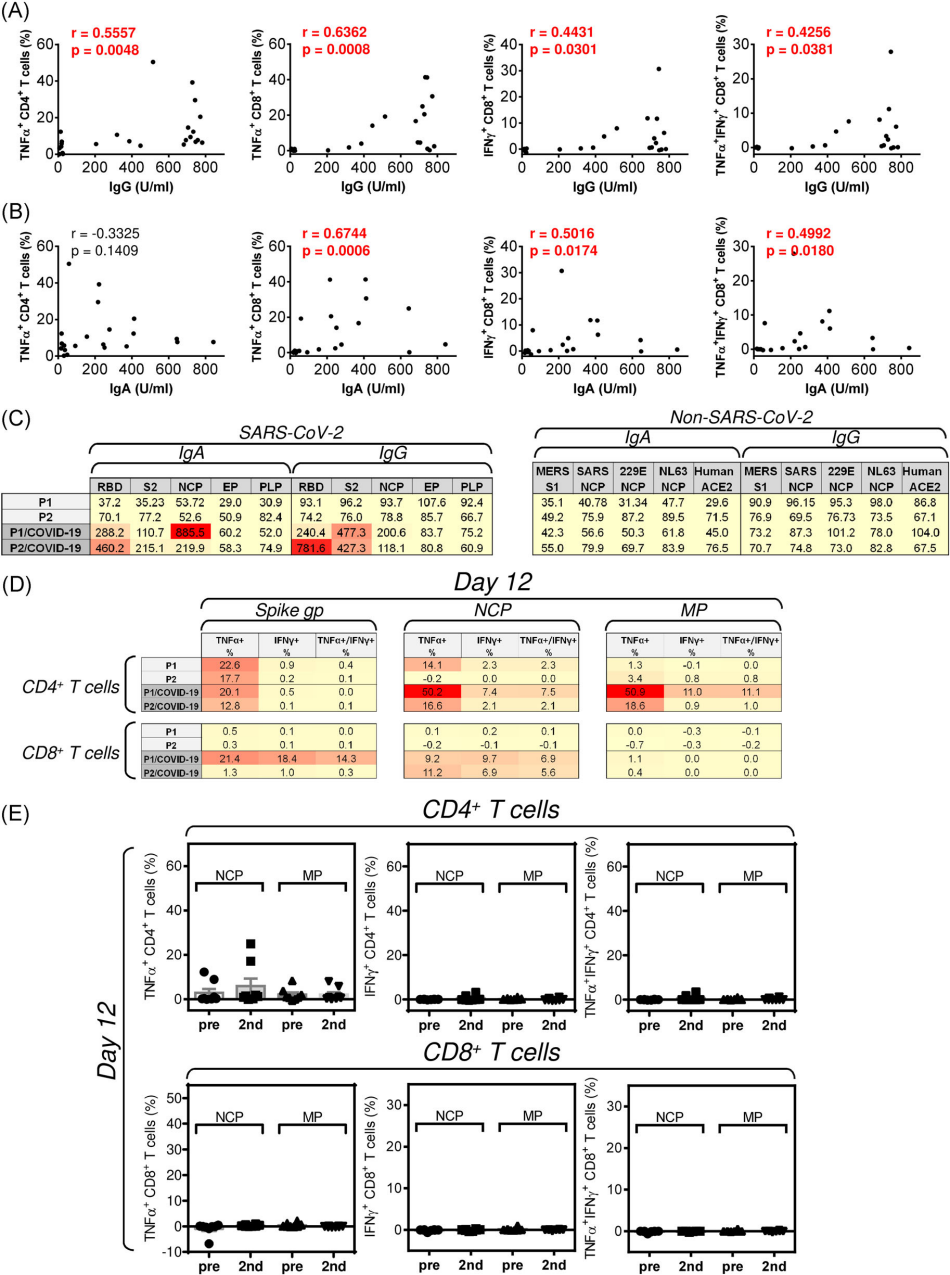
The association between the humoral response and T cell reactivity during the vaccination and the humoral and cellular responses to SARS‐CoV‐2 nucleocapsid protein and membrane protein. (A, B) The correlations between the levels (U/ml) of SARS‐CoV‐2 spike glycoprotein receptor‐binding domain (RBD)‐specific antibodies and the proportions of SARS‐CoV‐2 spike glycoprotein receptor‐reactive TNF‐α‐producing CD4^+^ and TNF‐α‐, IFNγ‐, or TNF‐α/IFNγ‐producing CD8^+^ T cells were evaluated by Spearman's rank‐order correlation coefficient (*r*) and the significance (*p* value) determined (IgA: *n* = 7 (HD1 was excluded because diagnosed as IgA deficient) and IgG: *n* = 8 healthy donors). (C) The serum levels of the antigen‐specific IgA and IgG, antibodies were determined by the Microblot‐Array COVID‐19 in two patients before COVID‐19 (P1 and P2) and 2 weeks after the COVID‐19 recovery (P1/COVID‐19 and P2/COVID‐19). In the two left panels, the serum levels (U/ml) of IgA and IgG antibodies are specific to SARS‐CoV‐2 proteins; spike glycoprotein receptor‐binding domain (RBD) and S2 domain (S2), nucleocapsid protein (NCP), E protein (EP), and papain‐like protease (PLP). In the two right panels, the serum levels (U/ml) of IgA and IgG antibodies are specific to non‐SARS‐CoV‐2 proteins; Middle East respiratory syndrome‐related coronavirus spike glycoprotein S1 subunit (MERS‐CoV, S1), SARS‐CoV nucleocapsid protein (SARS‐CoV, NCP), human coronavirus 229E NCP (HuCoV 229E, NCP), and human angiotensin‐converting enzyme (Human, ACE‐2). The samples with (U/ml) values < 185 were negative, between 185 and 210 borderlines, and >210 positive. (D, E) Reactivity of cell cultures enriched for 12 days with peptides derived from SARS‐CoV‐2 spike glycoprotein, nucleocapsid protein, or membrane protein. (D) PBMCs isolated from two patients before COVID‐19 (P1 and P2) and 2 weeks after the COVID‐19 recovery (P1/COVID‐19 and P2/COVID‐19) were enriched for 12 days with SARS‐CoV‐2 spike glycoprotein (Spike gp)‐, nucleocapsid protein (NCP)‐, or membrane protein (MP)‐derived peptides. The proportions of TNF‐α‐, IFNγ‐, or TNF‐α/IFNγ‐producing CD4^+^ and CD8^+^ T cells reactive to pertinent peptides were determined by ICS as in Figure 3. (E) PBMCs isolated from eight healthy donors (HDs) before the vaccine dose (pre) and 3–4 weeks after the second vaccine dose (2nd) were enriched for 12 days with SARS‐CoV‐2 nucleocapsid protein (NCP)‐ or membrane protein (MP)‐derived peptides. The proportions of TNF‐α‐, IFNγ‐, or TNF‐α/IFNγ‐producing CD4^+^ and CD8^+^ T cells reactive to pertinent peptides were determined by ICS as in Figure 3. The bars represent the mean of values ± *SEM* and significances of differences between the groups (Pre, 2nd) for TNF‐α‐, IFNγ‐, or TNF‐α/IFNγ‐producing CD4^+^ and CD8^+^ T cells are indicated (*p < .05, *n* = 8 HDs, Wilcoxon matched‐pairs signed‐ranks test)

Our data showed that the COVID‐19 vaccine promoted both humoral and cellular immunity against SARS‐CoV‐2 spike glycoprotein. We next analyzed whether the extent of the humoral response correlated with the ability of PBMCs to become enriched with SARS‐CoV‐2 spike glycoprotein‐reactive CD4^+^ and CD8^+^ T cells. As shown in Figure 4A and 4B, the extent of the humoral response correlated with the ability of PBMCs to become enriched with SARS‐CoV‐2 spike glycoprotein‐reactive CD4^+^ and CD8^+^ T cells. The levels of the RBD‐specific IgG antibodies were found to correlate with the extent of the PBMCs' ex vivo enrichment with the SARS‐CoV‐2 spike glycoprotein‐reactive TNF‐α‐producing CD4^+^ and TNF‐α‐, IFNγ‐, or TNF‐α/IFNγ‐producing CD8^+^ T cells (Figure 4A). Comparable data were obtained upon the correlations with the levels of RBD‐specific IgA antibodies (Figure 4A). The only exception was with no correlation found for the TNF‐α‐producing CD4^+^ T cells (Figure 4B, left panel). Overall, the data showed a close association between the COVID‐19 vaccination‐induced humoral and cellular responses against the SARS‐CoV‐2 spike glycoprotein.

### 3.4 COVID‐19 vaccination does not induce a humoral or cellular response to SARS‐CoV‐2 nucleocapsid and membrane protein

The infection with SARS‐CoV‐2 induces humoral immune responses to other viral proteins, including the NCP and, to a lesser extent, the membrane protein.^24^ The infection also induces a cellular response to these proteins,^25^ and these responses can be ex vivo enhanced.^9^, ^10^ We confirmed reactivity to these proteins by analyzing samples from two control donors (patients). Samples from these control donors (patients) were first collected before they contracted the virus and became sick with COVID‐19, and then 2 weeks after their recovery from the disease. As shown in Figure 4C, their sera before COVID‐19 contained no detectable antibodies against SARS‐CoV‐2 proteins. However, after COVID‐19, antibodies to one or more proteins of SARS‐CoV‐2 were already present in their sera, confirming the disease‐elicited humoral response against multiple proteins of the virus (Figure 4C, left panel). The specificity of the disease‐elicited humoral response was corroborated by the absence of non‐SARS‐CoV‐2‐specific antibodies in the post‐COVID‐19 sera (Figure 4C, right panel).

The cellular immune response to SARS‐CoV‐2 was more patient‐ and protein‐specific. As shown in Figure 4D, the disease promoted ex vivo enrichment with nucleocapsid and membrane protein‐reactive CD4^+^ T cells (Figure 4D, top middle and right panels). No promotion was observed for the spike glycoprotein‐reactive CD4^+^ T cells (Figure 4D, top left panel). The disease also did not promote enrichment with membrane protein‐reactive CD8^+^ T cells (Figure 4D, bottom right panel). However, the disease promoted strong enrichment with NCP‐reactive CD8^+^ T cells (Figure 4D, bottom middle panel). In one donor, a strong promotion was also found for the spike glycoprotein‐reactive CD8^+^ T cells (Figure 4D, bottom left panel). In the second donor, this promotion was much weaker (Figure 4D, bottom left panel). Unlike the disease, the COVID‐19 vaccination promoted no significant enrichment with nucleocapsid or membrane protein‐reactive CD4^+^ and CD8^+^ T cells (Figure 4E), confirming the vaccine's precision in eliciting specific immune responses against the target antigen.

### 3.5 The number of ex vivo‐enriched SARS‐CoV‐2 spike glycoprotein‐reactive CD4^+^ and CD8^+^ T cells can be large‐scale expanded in the cell culture

**Figure 5:**
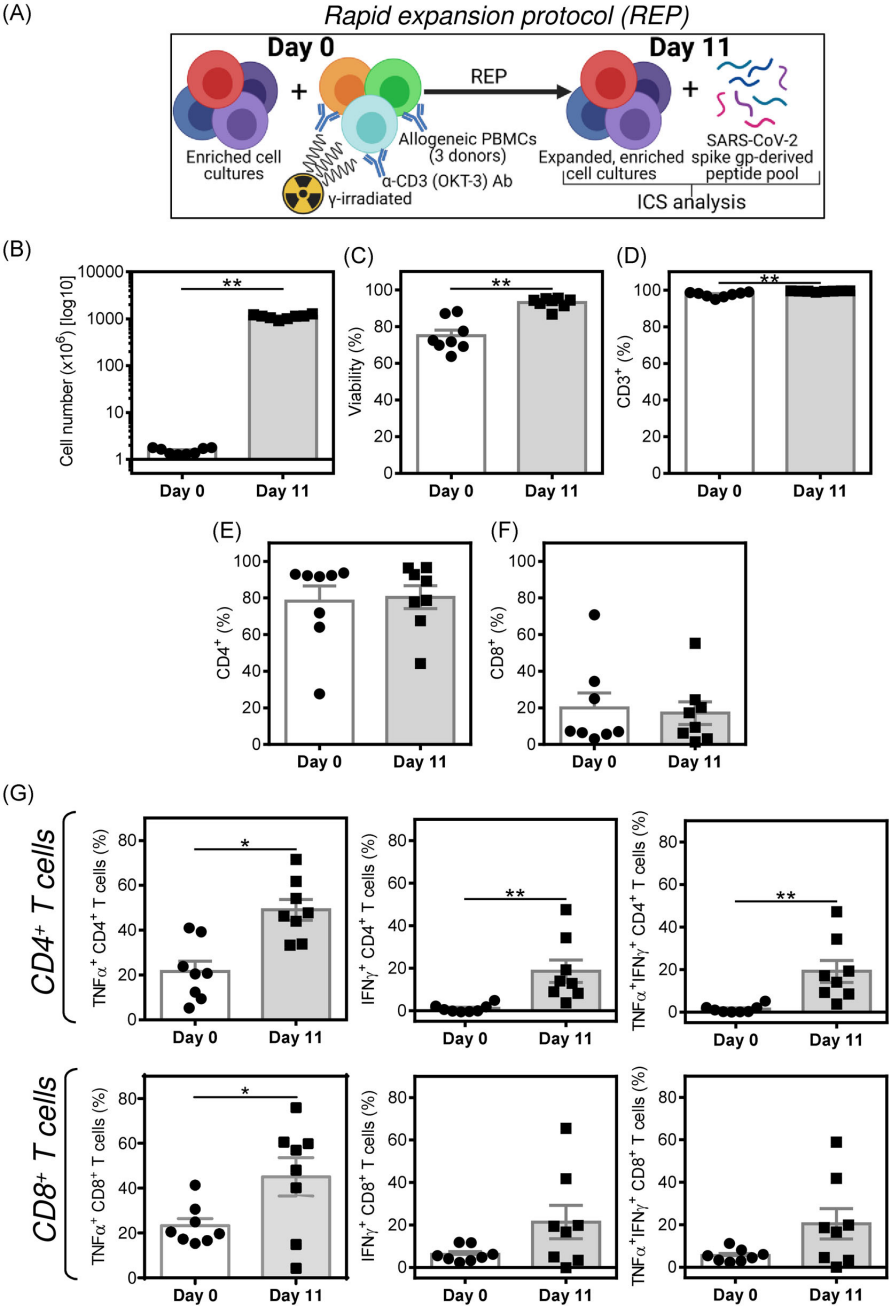
Large‐scale expansion of the peptide‐enriched cell cultures from the second dose‐vaccinated donors using rapid expansion protocol (REP). (A) Schematic presentation of REP and the intracellular cytokine staining (ICS) analysis. (B–F) Cell numbers before (Day 0) and after (Day 11) the REP (B), their viability (C), the proportion of T cells (CD3^+^) (D), and proportions of CD4^+^ (E) and CD8^+^ (F) T cell populations. (G) The peptide‐enriched (Day 0) and their REP‐expanded counterpart (Day 11) were, or not, stimulated with SARS‐CoV‐2 spike glycoprotein‐derived peptides and the proportions of TNF‐α‐, IFNγ‐, or TNF‐α/IFNγ‐producing CD4^+^ and CD8^+^ T cells determined by ICS. The proportions of reactive T cells were calculated as the difference between the proportions of the cytokine‐producing T cells of the vehicle‐stimulated sample and the peptide‐stimulated sample of the same donor. In (B–G), the bars represent the mean of values ± *SEM* and significances of differences among the groups (Days 0 and 11) for TNF‐α‐, IFNγ‐, or TNF‐α/IFNγ‐producing CD4^+^ and CD8^+^ T cells are indicated (*p < .05, **p < .01, *n* = 8 second dose‐vaccinated healthy donors, Wilcoxon matched‐pairs signed‐ranks test)

The peptide‐enrichment experiments showed that COVID‐19 vaccination could significantly enhance or even induce the PBMC's ability to become enriched with SARS‐CoV‐2 spike glycoprotein‐reactive CD4^+^ and CD8^+^ T cells. We further investigated whether this enrichment could also have the potential to become an avenue for a T cell‐based immunotherapy of COVID‐19. We used the peptide‐enriched cell cultures from the donors' PBMCs after the second dose of the vaccine and expanded the number of cells using the rapid expansion protocol (REP)^15^ (Figure 5A). As shown in Figure 5B, the 11‐day REP led to a 743.6 (range from 566.3 to 912.0, *n* = 8, 95% confidence interval [CI] = 632.0–855.2) cell number fold increase. The expanded cells were of higher viability, with increased proportions of T cells and similar proportions of CD4^+^ and CD8^+^ T cell populations (Figure 5C–F). Importantly, the expanded cell cultures became further enriched with the peptide‐specific CD4^+^ and CD8^+^ T cells (Figure 5G). As shown in Figure 5G, the enrichment was significant for both the peptide‐reactive CD4^+^ T cells producing TNF‐α‐, IFNγ‐, or TNF‐α/IFNγ and CD8^+^ T cells producing TNF‐α.

**Figure 6:**
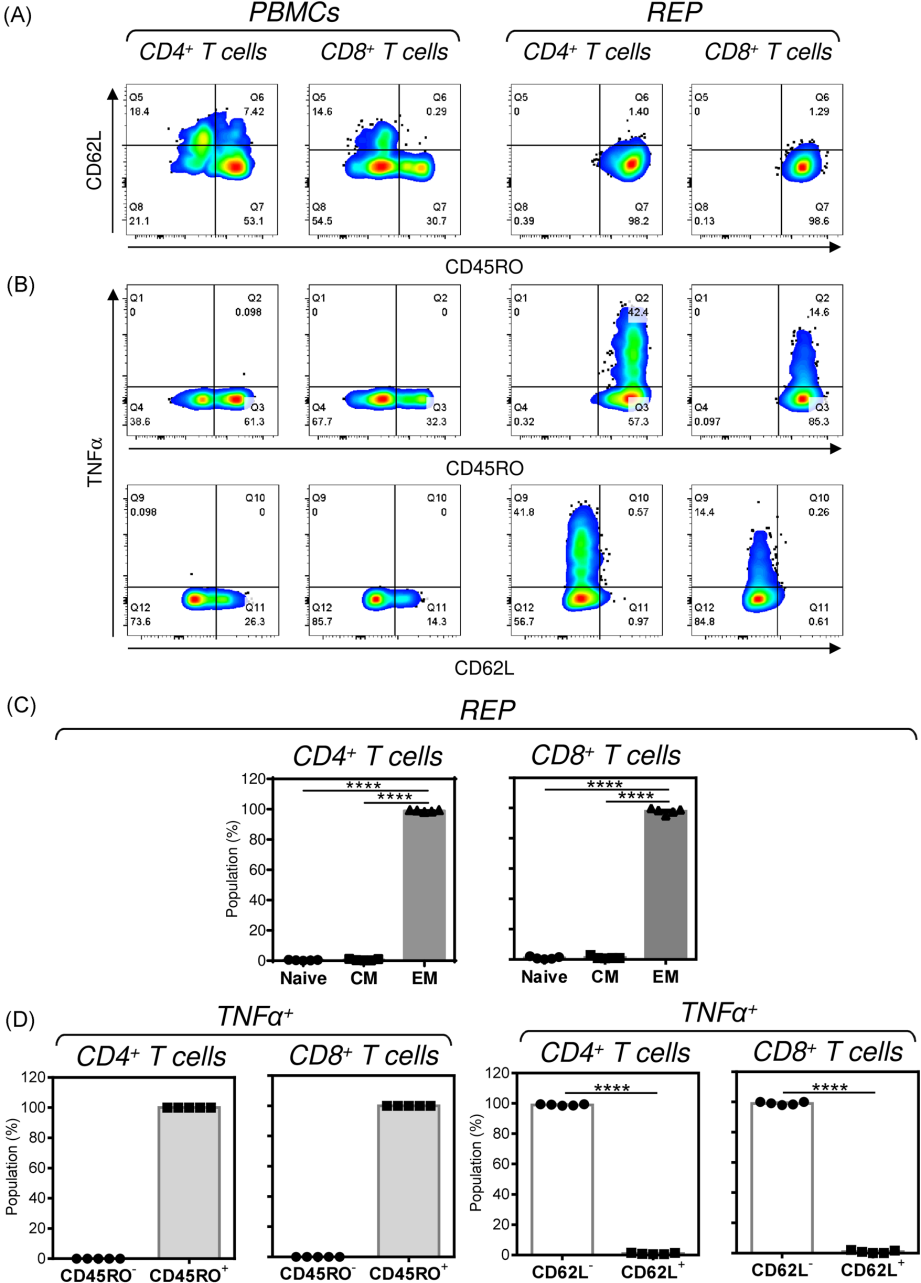
T cell phenotype of REP‐expanded T cells. (A) The REP‐expanded T cells (Day 11) were stimulated with SARS‐CoV‐2 spike glycoprotein‐derived peptides and the cells were fixed, permeabilized, and stained with live/dead fixable stain and CD3^−^, CD4^−^, CD8^−^, CD45RO^−^, CD62L^−^, and TNF‐α‐specific antibodies. CD4^+^ and CD8^+^ T cell were gated as in Figure 2A. The CD45RO and CD62L gating of T cells was performed using freshly isolated PBMCs as controls (two left panels). Shown are representative images. (B) CD45RO, CD62L, and TNF‐α gating of the peptide‐stimulated (two right panels) or non‐stimulated (two left panels) CD4^+^ and CD8^+^ T cells. Shown are representative images. (C) The proportions of naive (Naive, CD45RO^−^CD62L^+^), central memory (CM, CD45RO^+^CD62L^+^), and effector memory (EM, CD45RO^+^CD62L^−^) T cells in (A). The bars represent the mean of values ± *SEM* (****p < .0001, *n* = 5, one‐way ANOVA with the Tukey's posttest). (D) The proportions of CD45RO^−/+^ (two left panels) or CD62L^−/+^ (two right panels) populations of TNF‐α‐producing CD4^+^ and CD8^+^ T cells in (A). The bars represent the mean of values (two right panels: ****p < .0001, *n* = 5, Student's *t*‐test)

Next, we investigated the phenotype of the REP‐expanded cells. Analyzing the expression of CD45RO and CD62L as the T cells differentiation markers,^26^ we found that over 95% of the expanded T cells had the effector memory (CD45RO^+^CD62L^−^) phenotype (Figures 6A and 6C). Once REP‐expanded cells were stimulated with the SARS‐CoV‐2 spike glycoprotein‐derived peptides, all the peptide‐reactive TNF‐α‐producing CD4^+^ or CD8^+^ T cells were CD45RO^+^ (Figures 6B, top right panels and 6D, two left panels). Also, nearly all these TNF‐α‐producing CD45RO^+^ cells were CD62L^−^ (Figures 6B, bottom right panels and 6D, two right panels), therefore showing the SARS‐CoV‐2 spike glycoprotein‐specific T cells as the effector memory T cells.

The findings showed that the combination of COVID‐19 vaccination, peptide‐mediated enrichment, and REP could lead to the production of therapeutically relevant numbers of SARS‐CoV‐2 spike glycoprotein‐reactive effector memory CD4^+^ and CD8^+^ T cells.

## 4 DISCUSSION

This study showed that COVID‐19 vaccines could elicit both a humoral and cellular response against the virus. Using the cell culture techniques and peptides derived from the virus antigen, the vaccine‐induced antigen‐reactive T cells can be ex vivo‐enriched and large‐scale‐expanded and as such represent a potential therapeutic tool for the enhancement of cellular immunity after COVID‐19 vaccination.

The previous reports have shown that the BNT162b2 vaccine potentiated both the humoral and cellular responses,^27^ and this potentiation was even observed after one dose of the vaccine.^28^ Our data confirmed that vaccination of healthy donors with this vaccine indeed induced a humoral immune response that led to the production of SARS‐CoV‐2 spike glycoprotein‐specific IgG and IgA antibodies. This response was highly specific as the vaccination induced no detectable antibodies specific to other SARS‐CoV‐2 proteins or proteins from other coronaviruses. These data, therefore, confirmed the precision of the vaccine‐based prophylactic immunotherapy.

Cellular immunity is the important layer of immune protection against viruses as it prevents virus amplification after infection.^29^, ^30^ The effector cells of this arm of immunity are primarily the cytotoxic CD8^+^ T cells.^6^ This study showed that no such SARS‐CoV‐2 spike glycoprotein‐specific CD8^+^ T cells were detected in the peripheral blood of either non‐vaccinated or two‐dose‐vaccinated donors. These CD8^+^ T cells were also not detectable in the non‐vaccinated donors even after the peptide‐mediated enrichment. These data were in line with our recent study where samples from SARS‐CoV‐2‐unexposed donors or prostate cancer patients were also largely not enriched with SARS‐CoV‐2 spike glycoprotein‐specific CD8^+^ T cells.^31^ Even though we cannot entirely exclude that after so many months of the raging pandemic, some of the healthy donors in this study had contracted SARS‐CoV‐2 unnoticed, then having COVID‐19 with no or mild symptoms,^9^, ^10^ the donors showed no detectable trances of the virus‐specific immunity before the COVID‐19 vaccination. Unlike the two control donors (patients) analyzed in this study, the healthy donors of this study had no detectable SARS‐CoV‐2 spike glycoprotein‐, NCP‐, or membrane protein‐specific antibodies in their sera, nor their PBMCs had the potential to become enriched with neither of the virus protein‐reactive T cells. However, once these healthy donors obtained two doses of the COVID‐19 vaccine, the peptide‐mediated enrichment already produced cell cultures containing the SARS‐CoV‐2 spike glycoprotein‐specific CD8^+^ T cells. The impact of the COVID‐19 vaccine was also specific because it did not promote the peptide‐mediated enrichment with SARS‐CoV‐2 nucleocapsid or membrane protein‐specific T cells. These data showed that the vaccination was important for increasing the frequency of the SARS‐CoV‐2 spike glycoprotein‐specific CD8^+^ T cells to the levels that allowed their peptide‐mediated enrichment in the cell culture. These findings corroborate previous reports showing increased frequencies of T cells reactive to peptides derived from the tumor‐associated antigens in the peptide‐enriched cell cultures after the patients' vaccination with ex vivo‐produced dendritic cells loaded with whole inactivated tumor cells.^32^, ^33^

Our results showed that humoral and T cell‐based immune responses went hand in hand in the tested healthy donors. However, patients with compromised immunity or undergoing therapies that compromise their immunity may not respond well with both arms of the adaptive immunity, and the protective potential of the COVID‐19 vaccines can then be undermined in these patients.^34^ The large‐scale expanded antigen‐specific T cells have been utilized for adoptive cellular immunotherapy (ACI) of cancer.^35^ Both prophylactic and therapeutic antiviral ACI have also been utilized after the hematopoietic stem cell (HSC) transplantations, where viral infections are an important cause of morbidity and mortality.^36^, ^37^ The restoration of the viral immunity is often successfully attained by the adoptive transfer of the HSC donor's ex vivo expanded virus‐specific CD8^+^ T cells.^36^, ^37^ The expanded SARS‐CoV‐2‐reactive T cells could, therefore, be also implemented in these therapeutic strategies to compensate for insufficiencies of the SARS‐CoV‐2‐specific cellular immunity. Previous studies have shown that therapeutically relevant numbers of SARS‐CoV‐2‐reactive T cells could be ex vivo large‐scale expanded from COVID‐19 convalescent donors.^9^, ^10^ The findings of this study showed that these therapeutic‐relevant numbers could also be attained after the vaccination of donors with no previous detectable virus‐specific immunity nor evidence of a previous COVID‐19. The findings of this study showed that the combination of the COVID‐19 vaccines with the ex vivo peptide‐mediated enrichment and large‐scale expansion could represent a viable approach for the production of T cells for cellular therapy of COVID‐19.

## CONFLICT OF INTERESTS

Jirina Bartunkova is a part‐time employee and a minority shareholder of Sotio, as Pavla Taborska, Jan Lastovicka, Dmitry Stakheev, Zuzana Strizova, Daniel Smrz declare no conflicts of interest.

## ETHICS STATEMENT

All experimental protocols were approved by the ethical standards of the institutional and/or national research committee – the Ethics Committee of the University Hospital Motol in Prague, and performed in accordance with the 1964 Helsinki declaration and its later amendments or comparable ethical standards. All patients provided signed informed consent for the use of their blood‐derived products for future research.

## AUTHOR CONTRIBUTIONS

Pavla Taborska, Jan Lastovicka, and Daniel Smrz conducted the experiments and/or analyzed the data; Pavla Taborska and Daniel Smrz designed the experiments; Dmitry Stakheev, Zuzana Strizova, and Jirina Bartunkova supervised the sample collection and clinical aspects of the study; Daniel Smrz wrote the manuscript; Pavla Taborska, Jan Lastovicka, Dmitry Stakheev, Zuzana Strizova, and Jirina Bartunkova contributed to the writing of the manuscript; Daniel Smrz supervised the research. Research in the authors' laboratories was supported by funding from Charles University PRIMUS/MED/12 – and funding from the Ministry of Health, Czech Republic – project AZV 16‐28135A.

## Data Availability

The raw data supporting the conclusions of this article will be made available by the authors, without undue reservation.
